# Comparison of the Asymmetries in Foot Posture and Properties of Gastrocnemius Muscle and Achilles Tendon Between Patients With Unilateral and Bilateral Knee Osteoarthritis

**DOI:** 10.3389/fbioe.2021.636571

**Published:** 2021-10-14

**Authors:** Zehua Chen, Xiangling Ye, Zhen Shen, Yi Wang, Zugui Wu, Guoqian Chen, Yingxin Guan, Jiatao Wu, Tao Jiang, Huai Wu, Wengang Liu, Xuemeng Xu

**Affiliations:** ^1^ The Fifth Clinical Medical College of Guangzhou University of Chinese Medicine, Guangzhou, China; ^2^ Kunming Municipal Hospital of Traditional Chinese Medicine, Kunming, China; ^3^ Department of Orthopaedic Surgery, Zhejiang Provincial Hospital of Chinese Medicine, Hangzhou, China; ^4^ The Second Affiliated Hospital of Guangzhou University of Chinese Medicine/Zhuhai Hospital of Guangdong Province Traditional Chinese Medical Hospital, Zhuhai, China; ^5^ Guangdong Second Traditional Chinese Medicine Hospital, Guangzhou, China

**Keywords:** knee osteoarthritis, gastrocnemius muscle, Achilles tendon, asymmetry, Foot Posture index, foot posture

## Abstract

**Background:** Asymmetrical foot posture and properties alterations of the gastrocnemius muscle (GM) and Achilles tendon (AT) were observed in knee osteoarthritis (KOA). We aimed to investigate the inter-limbs asymmetries in foot posture and the properties of GM and AT and explore the association between them.

**Methods:** A total of 62 subjects with unilateral or bilateral KOA were included in this study: 30 patients with unilateral pain and 32 patients with bilateral pain were assigned to the bilateral group (BG) and unilateral group (UG), respectively. The relatively serious leg (RSL) and relatively moderate leg (RML) were judged according to the severity of symptoms assessed by using visual analogue scale (VAS) motion. Foot posture and asymmetrical foot posture scores were assessed based on Foot Posture index (FPI-6). Subsequently, all the participants received an assessment for properties of GM and AT, including tone/tension (Hz), stiffness (N/m), and elasticity. We calculated the asymmetry index of AT (Asy_*-*AT_) in both legs and the difference of muscle properties between medial and lateral gastrocnemius (D_-MLG_) in the ipsilateral limb.

**Results:** Asymmetry of foot posture was categorized into three types including normal, asymmetry, and severe asymmetry. The percentage of subjects classified as normal was higher in the BG (62.5%) than in the UG (36.67%), *p* < 0.05. Tension of AT and tone of lateral gastrocnemius (LG) in RSL were higher than those in RML (15.71 ± 0.91 vs. 15.23 ± 1.01; 25.31 ± 2.09 vs. 23.96 ± 2.08, *p* < 0.01 and *p* < 0.01, respectively), and stiffness of AT in the RSL was higher than that in RML (676.58 ± 111.45 vs. 625.66 ± 111.19, *p* < 0.01). Meanwhile, a positive relationship was found between ipsilateral FPI and tone of MG and LG in the left leg (0.246 per degree, 95% CI: −0.001, 0.129; *p* = 0.054 and 0.293 per degree, 95% CI: −0.014, 0.157; *p* = 0.021, respectively) and right leg (0.363 per degree, 95% CI: 0.033, 0.146; *p* = 0.004 and 0.272 per degree, 95% CI: 0.007, −0.144; *p* = 0.032, respectively). Moreover, a positive link was observed between asymmetrical FPI scores and K/L grade (0.291 per degree, 95% CI: 0.018, 0.216; *p* = 0.022). Furthermore, a significantly greater Asy_-AT(tension)_ was detected in the UG than that in the BG (UG vs. UG: 8.20 ± 5.09% vs. 5.11 ± 4.72%, *p* < 0.01). Additionally, an increased asymmetrical FPI score (i.e., more severe asymmetry) was significantly associated with increases in Asy_-AT(tension)_ and Asy_-AT(stiffness)_ (0.42 per degree, 95% CI: 0.533, 1.881; *p* = 0.001 and 0.369 per degree, 95% CI: 0.596, 2.82; *p* = 0.003, respectively).

**Conclusions:** The stiffness and tension of AT and the tone of LG in RSL were higher than those in RML in KOA patients, and inter-limbs foot posture and tension of AT were more asymmetrical in unilateral KOA patients compared to patients with bilateral KOA. Notably, foot posture, as an important biomechanical factor, was significantly associated with properties of GM, AT, and K/L grade in KOA patients.

## Introduction

With the growing population of aging and obesity, more and more people will experience knee osteoarthritis (KOA), exhibiting many severe symptoms, such as pain, joint swelling, dysfunction, and even limb deformity, which will heavily compromise the quality of people’s life (Hunter and Bierma-Zeinstra, 2019). In order to solve the serious public health problem, continuous exploration of mechanism researches has been conducted. As a multi-joint affected disease, KOA is found to be associated with biomechanical alterations of the adjacent joints (Gonçalves et al., 2017), and changes from ankle/foot are considered as potential determinants contributing to KOA.

Foot posture index (FPI) (Redmond et al., 2008) can effectively evaluate foot posture and categorize feet into three types: pronated, neutral, and supinated position, and it has been proven to be a reliable, simple, and economical method compared to conventional measures (Evans et al., 2003), footprints (Gijon-Nogueron et al., 2020), and automatic measurement apparatus (Ohi et al., 2017). Previous studies have suggested that foot posture evaluated by FPI was closely associated with pain, function in KOA patients (Al-Bayati et al., 2018), and a more pronated foot was found in the patients with medial KOA than in healthy subjects (Abourazzak et al., 2014). Moreover, some evidence has suggested that frontal plane knee alignment was associated with calcaneus angle (Ohi et al., 2017).

It has been reported that there are some inter-limb asymmetries in KOA. In people suffering from KOA, knee pain appeared to be associated with asymmetries in knee biomechanics (Creaby et al., 2012), and leg muscle asymmetry was more commonly presented in patients with higher radiographic grade and prevalent knee pain (Lee et al., 2019). Moreover, in their cohort study, Creaby et al. (Creaby et al., 2012) have highlighted that asymmetry of knee flexion moment was observed in patients with unilateral KOA, whereas the knee varus-valgus angle was symmetric in bilateral KOA patients. In our previous study (Chen et al., 2020), we have found that foot posture asymmetry in KOA patients was more severe than in healthy individuals. However, until now, foot posture asymmetry in patients with symptomatic KOA in the bilateral and unilateral knee has been scarcely reported.

Gastrocnemius muscle (GM) was considered to be closely associated with KOA. It has been previously shown that there was a significant correlation between AT thickness and KOA severity (Elbaz et al., 2017), and a diminished phase shift was detected between medial and lateral gastrocnemius activation in severe KOA patients during walking (Rutherford et al., 2011). GM is one of the knees’ spanning flexor muscles, of which activation could affect knee adduction moment (Booij et al., 2020), which was a reliable and accurate measurement of load exerted on the medial compartment of the knee joint, and had been proven to be highly related to the KOA severity (Khan et al., 2018). Meanwhile, it was suggested that foot posture was related to the foot center of pressure and could affect the knee adduction moment by the mediolateral center of pressure shift (Solomonow-Avnon et al., 2019), subsequently exerting an impact on KOA. Furthermore, there was an interaction between foot posture and alterations of Achilles tendon (AT) and GM. On the one hand, AT and GM, surrounding the joint, potentially vary with foot/ankle position due to the effects of foot posture on ankle mechanics. In the previous study, Ashnagar et al. (2019a) have found that there were some differences in thicknesses of leg muscles in KOA patients with different foot postures. On the other hand, contracture of GM would lead to alterations of foot posture. Myerson (2014) has indicated that a lack of dorsiflexion in the ankle induced by GM contracture might be compensated by dorsiflexion through foot pronating, and there would be a secondary effect on the Achilles as the heel moves into valgus in return.

As reported, the asymmetrical foot posture was observed in KOA patients. Long-term asymmetry foot posture would cause imbalance mechanics in two limbs, and the adjacent muscles of the ankle joint should be most easily affected, which might result in some alterations of properties of GM and AT. These properties can be measured using a non-invasive digital palpation device that can accurately assess the properties of superficial muscle and tendon, including tone, stiffness, and elasticity, which reflect the tissue condition objectively (Chang et al., 2020), representing their tissue properties (Feng et al., 2018), function, power, and locomotive efficiency (Gavronski et al., 2007). Considering interactions among AT, GM, foot posture, and KOA, we have hypothesized that there might be a correlation between foot posture asymmetry and muscle properties of AT and GM in KOA patients. In the light of the above-mentioned knowledge, the purpose of this study was to observe the inter-limbs asymmetries in foot posture and the properties of GM and AT in people with bilateral and unilateral symptomatic KOA and explore the relationship between foot posture asymmetry and properties of AT and GM, which would provide references for further clinical practice.

## Methods

### Study Design

This cross-sectional study was carried out at the Guangdong Second Traditional Chinese Medicine Hospital from September 2019 to February 2020. Ethical approval was granted by the Ethics Committee of Guangdong Second Traditional Chinese Medicine Hospital (No. E1923) and registered at the China Clinical Registration Center (Registration No. ChiCTR1900026400). In this study, all included participants provided written informed consent and could withdraw from the study at any time.

### Participants

All the participants were recruited from outpatients of the hospital. The inclusion criteria were 1) age> 45 years and KOA diagnosed by the American College of Rheumatology clinical criteria (Altman et al., 1986), 2) Kellgren/Lawrence (Kellgren and Lawrence, 1957) (K/L) grade ≥2 in one or two knees, 3) presence of predominantly medial compartment OA, and 4) an ability to stand independently on the platform without any assistive device. The exclusion criteria were 5) any known inflammatory arthritis, 6) concomitant neurologic diseases, such as stroke, 7) congenital or traumatic lower limb deformity/length discrepancy, 8) history of ankle diseases and lower extremity fracture/surgery, 9) any medication that affects properties of muscle and tendon, and 10) any vigorous exercise within 48 h of the test. In this study, lateral KOA patients were excluded because of the higher prevalence of the medial type in China (Sun et al., 2019). A total of 62 subjects with unilateral or bilateral KOA were included: 30 patients with unilateral pain and 32 patients with bilateral pain were divided into the unilateral group (UG) and bilateral group (BG). According to the severity of symptoms assessed using the visual analogue scale (VAS) motion, the symptomatic leg (or the most symptomatic leg in a case of bilateral involvement) was defined as the relatively serious leg (RSL) and the contralateral side as the relatively moderate leg (RML). If KOA patients experienced the same symptomatic pain in both legs, we would define the RSL and RML through a randomized method (toss a coin).

### Evaluation of the Foot Posture

Foot posture was evaluated on both feet using Foot Posture Index-6 (Redmond, 2015) (FPI-6). According to the FPI total score, feet were classified as three types: neutral (from 0 to +5), pronated (greater than or equal to 6), and supinated (less than or equal to −1). Based on the previous method (Chen et al., 2020), prior to standing still, participants were asked to march on the spot. During the assessment period, they stood barefoot in bilateral support with their arms by the side and looking straight ahead to avoid changes in foot posture caused by swiveling. The assessment was conducted by two investigators (ZH.C and XL.Y), and excellent inter-observer and intra-observer reliability of FPI scores were observed using the intraclass correlation coefficient in our previous study (ICC: 0.926 and 0.946, respectively) (Chen et al., 2020).

### Evaluation of the Foot Posture Asymmetry

Foot posture asymmetry was classified according to the asymmetry score (difference in FPI score between the two feet) based on the previous method (Rokkedal-Lausch et al., 2013), which was calculated as FPI score on the right foot minus the FPI score on the left foot. Asymmetry score ranging from −2 to +2 represented normal, −4 ≤ asymmetry score <−2 or +2 < asymmetry score ≤+4 was asymmetry, and severe asymmetry was <−4 or >4.

### Evaluation of Properties of Achilles Tendon and Gastrocnemius Muscle

Properties of AT and GM were determined using a non-invasive handheld machine (MyotonPRO, Estonia, serial number: 000297, product manufacturer code: 1308600502). According to the previous studies (Huang et al., 2018; Morgan et al., 2018), the measurement sites of the medial of the gastrocnemius (MG) and lateral of the gastrocnemius (LG) were located at 70% of the lower leg length measured from the lateral malleolus to the popliteal fossa and at 1/3 of the leg length from the fibular head to the heel, respectively, and AT was measured at 6 cm from bone insertion (Supplementary Figure S1). The assessments were conducted in the following order: AT (left), AT (right), MG (left), MG (right), LG (left), and LG (right). During the testing, the participants were required to lie prone on a couch with feet and legs exposed in a fully relaxed position. One researcher (GQ. C) helped to keep the knee fully extended and the ankle at 45° plantar flexion measured by a protractor. Another researcher (T. J) held the MyotonPRO perpendicular to the skin surface. Pushed against the tested area with a 0.18 N prepressure, after the red light turned green, five short mechanical impulses (0.4 N, tap interval was 0.8 s) were generated automatically by the device to induce oscillations in the soft tissues. The parameters recorded and computed automatically included oscillation frequency (Hz), reflecting tone/tension; dynamic stiffness (N/m); logarithmic decrement (log decrement), characterizing elasticity; creep (C); mechanical stress relaxation time (ms) (Myoton.com). The two examiners were blinded to the group allocation. We set the procedure as follows: five short impulses (tap interval was 0.8 s) to induce mechanical oscillations (Chen et al., 2019). The measurement was repeated three times and averaged, and an excellent inter-observer and intra-observer reliability was observed in our previous study (Chen et al., 2019).

We calculated the differences of muscle properties between MG and LG (D_-MLG_) in the ipsilateral limb using the larger value minus the lower value, and D_-MLG(tone)_, D_-MLG(stiffness)_, and D_-MLG(elasticity)_ were used to represent the difference of tone, stiffness, and elasticity between the MG and LG in one leg. In addition, AT asymmetry index (Asy_*-*AT_) in two legs was also calculated (Supplementary Method S1) in accordance with the previous study (Iijima et al., 2019). Similarly, Asy_-AT(tension)_, As_-AT(stiffness)_, and Asy_-AT(elasticity)_ were used to represent the Asy_-AT_ index of tone, stiffness, and elasticity in both legs.

### Statistical Analysis

We calculated the sample size using PASS 15.0.5. To evaluate the difference in the percentage of FPI asymmetry, when power = 0.8/0.85, the sample size should not be less than 50(25/25)/58(29/29). For the difference in the continuous variable, the sample size should be larger than 46 (power = 0.9, 23/23) for non-paired data or 56 (power = 0.85, 28/28) for paired data. In this study, linear regression or multiple linear regression was used to examine the correlations. When evaluating the correlations, all the included patients, comprising unilateral and bilateral KOA patients, were merged. As the power = 0.9, the minimum sample size according to linear regression and multiple linear regression should be 34 and 48, respectively. All statistical analyses were conducted using SPSS25.0 (IBM Corp., NY, United States) software. Continuous characteristics of the study were checked for normality using the Shapiro–Wilk test. Comparing the parameters between MG and LG, or two legs, in the same patients, the paired Student *t*-test was used for normal distribution, or the Wilcoxon test was selected. An independent *t*-test or non-parametric test (Mann–Whitney) was used to assess the differences between two groups according to the result from the previous normality test. A chi-square test was performed to examine between-group comparison categorical variables. Multiple linear regression expressed as regression coefficients (betas) and their 95% confidence intervals was calculated to determine the correlation analysis between foot posture parameters, including FPI score and absolute values of asymmetry score, and muscle properties. For multiple linear regression analysis, the foot posture parameters were defined as the independent variable and the muscle properties were set as a predictor. All continuous variables were presented as mean ± standard deviations. Statistical significance level was accepted at *p* < 0.05.

## Results

### Participants Characteristics

In this study, 62 patients were included according to the inclusion and exclusion criteria in the final analysis. Participants’ characteristics and foot morphology are listed in Table 1. No significant difference was observed in their age, weight, height, and BMI between the UG (30 patients) and BG (32 patients).

**TABLE 1:** Demographic characteristics and foot posture of the study patients.

	UG (*n* = 30)	BG (*n* = 32)	Merged
Age (years)	62.97 ± 6.96	60.09 ± 6.12	61.48 ± 6.65
Female/Male	13/17	15/17	28/34
Height (cm)	156.13 ± 27.20	161.84 ± 6.27	159.08 ± 19.50
Weight (kg)	63.30 ± 8.42	64.44 ± 7.40	63.89 ± 7.86
BMI (kg/m^2^)	24.35 ± 2.80	24.57 ± 4.51	24.46 ± 2.46
K/L grade	3.03 ± 0.72	2.72 ± 0.68	2.87 ± 0.71
Ⅱ	7	13	20
Ⅲ	15	15	30
Ⅳ	8	4	12
RSL (left/right)	18/12	16/16	34/28
RML (left/right)	12/18	16/16	28/34
**FPI score**			
Left	4.53 ± 3.59	3.22 ± 3.45	3.85 ± 3.55
Right	4.63 ± 3.77	3.44 ± 3.37	4.02 ± 3.59
RSL	4.87 ± 3.44	3.31 ± 3.20	4.06 ± 3.38
RML	4.30 ± 3.89	3.34 ± 3.62	3.81 ± 3.75
FPI asymmetry score	0.10 ± 3.84	0.22 ± 2.85	0.16 ± 3.34

UG: unilateral group; BG: bilateral group; BMI: body mass index; RSL: relatively severe leg; RML: relatively moderate leg.

### Foot Posture Asymmetry Analysis

**FIGURE 1:**
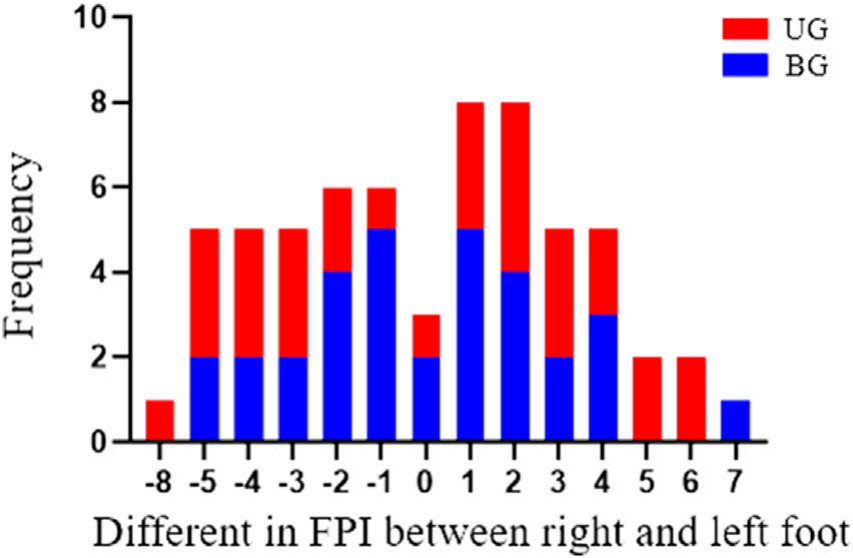
The number of subjects with various FPI asymmetry scores in BG and UG. UG: unilateral group; BG: bilateral group.

As was illustrated in Figure 1, asymmetry score, difference in FPI between right foot and left foot, mainly centered on −2 to 2 in the BG, whereas it distributed relatively wide in UG. According to asymmetry score, the patients in the two groups were categorized into three types: normal, asymmetry and severe asymmetry. It was detected that the percentage of subjects classified as the normal was higher in the BG (62.5%) than the UG (36.67%), and the ratio of asymmetric total (asymmetry and severe asymmetry) was lower in the BG (37.5%) than in the UG (63.33%), *p* = 0.042 (Table 2).

**TABLE 2:** Comparison of FPI asymmetry score between two groups.

Groups	FPI asymmetry score		
	Normal	Asymmetry	Severe asymmetry
BG	20 (62.5%)^*^	9 (28.13%)	3 (9.38%)
UG	11 (36.67%)	11 (36.67%)	8 (26.67%)

UG: unilateral group; BG: bilateral group; *Compared to BG, *p* < 0.05.

### Gastrocnemius Muscle and Achilles Tendon Properties Analysis

**FIGURE 2:**
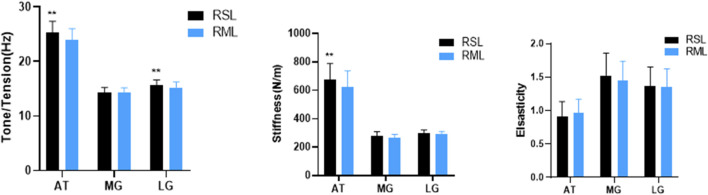
Comparison of GM and AT properties between RSL and RLL. GM: gastrocnemius muscle; MG: medial of gastrocnemius; LG: lateral of gastrocnemius; RSL: relatively severe leg; RML: relatively moderate leg; AT: Achilles tendon; compared to RML, **p < 0.05.

Tension of AT and tone of LG in RSL was higher than RML (*p* < 0.01 and *p* < 0.01, respectively). The stiffness of AT in the RSL was larger than that in the RML (*p* < 0.01). However, for elasticity, there was no significant difference in AT, MG, and LG between RSL and RML (shown in Figure 2). Meanwhile, AT presented a significantly greater Asy_-AT(tension)_ in the UG than that in the BG (*p* < 0.01), whereas the difference in Asy_-AT(stiffness)_ and Asy_-AT(elasticity)_ failed to reach statistical significance in AT, *p* = 0.205 and *p* = 0.197, respectively. The corresponding data are shown in Table 3. Furthermore, compared to LG, the tone and stiffness of MG were lower in both left and right leg, whereas elasticity was significantly larger (Supplementary Figure S2). D_-MLG_ (difference of muscle properties between the MG and LG in the ipsilateral limb), including D_-MLG(tone)_, D_-MLG(stiffness)_, and D_-MLG(elasticity)_, did not differ significantly between RHL and RLL (Supplementary Table S1).

**TABLE 3:** Comparison of Asy_-AT_ between UG and BG.

Groups	Asy_-AT_		
	Asy_-AT(tension)_ (%)	Asy_-AT(stiffness)_ (%)	Asy_-AT(elasticity)_ (%)
UG	8.20 ± 5.09^**^	12.56 ± 8.76	21.24 ± 16.34
BG	5.11 ± 4.72	9.52 ± 8.04	16.07 ± 12.09

AT: Achilles tendon; Asy_-AT_: asymmetry index of Achilles tendon; Asy_-AT(tension)_: asymmetry index of tone; Asy_-AT(stiffness):_ asymmetry index of tone; Asy_-AT(elasticity)_: asymmetry index of elasticity; UG: unilateral group; BG: bilateral group. Compared to BG, **p < 0.01.

### Correlations Between FPI Scores and Gastrocnemius Muscle and Achilles Tendon Properties

Multiple linear regression revealed the association between FPI scores and GM and AT properties, and the results are shown in Table 4. Increased FPI score was correlated to the tone and stiffness of MG in the right side (0.363 per degree, 95% CI: 0.033, 0.146; *R* ^2^ = 0.132; *p* = 0.004 and 0.28 per degree, 95% CI: 0.256, 4.263; *R* ^2^ = 0.132; *p* = 0.028, respectively), and a similar trend of near correlation to statistically significant was observed between them in the left side (0.246 per degree, 95% CI: −0.001, 0.129; *R* ^2^ = 0.061; *p* = 0.054 and 0.249 per degree, 95% CI: −0.008, 4.009; *R* ^2^ = 0.062; *p* = 0.051, respectively). A positive relationship was detected between ipsilateral FPI and tone of LG in both left and right legs (*p* = 0.021 and 0.032 respectively). Morever, there was a positive link between asymmetrical FPI scores and K/L grade (*p* = 0.022). As shown in Table 4, an increased asymmetrical FPI score (i.e., more severe asymmetry in both feet) was significantly associated with the increases in Asy_-AT(tension)_ (0.42 per degree, 95% CI: 0.533, 1.881; *R* ^2^ = 0.176; *p* = 0.001) and Asy_-AT(stiffness)_ (0.369 per degree, 95% CI: 0.596, 2.82; *R* ^2^ = 0.136; *p* = 0.003), whereas the remaining results did not show significant difference.

**TABLE 4:** The association of FPI scores with GM and AT properties.

Dependent variables	FPI (left)	FPI (right)	Asymmetrical FPI score	
**Beta (95% CI) of MG, per degree`**				
Tone	♠0.246 (−0.001, 0.129)	♣0.363 (0.033, 0.164)^**^		
Stiffness	♠0.249 (−0.008, 4.009)	♣0.280 (0.256, 4.263)^*^		
Elasticity	♠0.101 (−0.014, 0.033)	♣−.072 (−0.029, 0.016)		
**Beta (95% CI) of LG, per degree**				
Tone	♠0.293 (−0.014, 0.157)^*^	♣0.272 (0.007, −0.144)^*^		
Stiffness	♠0.152 (−0.707, 2.775)	♣0.127 (−3.103, 0.649)		
Elasticity	♠−0.034 (−0.021, 0.016)	♣0.080 (−0.015, 0.029)		
**Beta (95% CI) of AT, per degree**				
Tension	♠0.136 (−0.183, 0.597)	♣0.075 (−0.119, 0.217)		
Stiffness	♠0.137 (−0.003, 0.011)	♣0.163 (−2.962, 13,405)		
Elasticity	♠−0.092 (−4.567, 2.165)	♣0.122 (−0.007, 0.02)		
**Beta (95% CI) of Asy_-AT_, per degree**				
Asy_-AT(tension)_ (%)	0.130 (−0.187, 0.571)	−0.02 (−0.354, 0.413)	0.420 (0.533, 1.881)^**^	
Asy_-AT(stiffness)_ (%)	−0.011 (−0.641, 0.590)	−0.14 (−0.946, 0.277)	0.369 (0.596, 2.82)^**^	
Asy_-AT(elasticity)_ (%)	−0.248 (−2.008, 0.008)	−0.296 (−2.196, 0.201)	0.211 (−0.322, 3.631)	
**Beta (95% CI) of D_-MLG_, per degree**				
D_-MLG(tone)_	♠ 0.092 (−0.045, −0.094)	♣ −0.163 (−0.101, 0.022)	♥ 0.113 (−0.076, 0.197)	♦ 0.078 (−0.083, 0.155)
D_-MLG(stiffness)_	♠ −0.106 (−2.397, 1.00)	♣ −0.107 (-2.088, 0.858)	♥ 0.208 (−0.510, 5.319)	♦ 0.290 (0.527, 6.683)
D_-MLG(elasticity)_	♠ 0.07 (−0.015, 0.027)	♣ −0.204 (−0.034, 0.004)	♥ −0.007 (−0.037, 0.037)	♦ −0.163 (−0.066, 0.015)
Asymmetrical FPI score	−0.200 (−0.838, 0.098)	−0.232 (−0.254, 0.01)		
K and L grade			0.291 (0.018, 0.216)^*^	

95% CI: 95% confidence interval; PG: patient group; CG: control group; FPI: Foot Posture index; AT: Achilles tendon; GM: gastrocnemius muscle, Asy_-AT(tension)_: asymmetry index of tone; Asy_-AT(stiffness):_ asymmetry index of tone; Asy_-AT(elasticity)_: asymmetry index of elasticity; D_-MLG:_ differences in muscle properties between medial and lateral gastrocnemius in the ipsilateral limb; D_-MLG(tone)_: difference in tone; D_-MLG(stiffness)_: difference in stiffness; D_-MLG(elasticity)_: difference in elasticity. ♠, ♣, ♥, and ♦ represented the correlation calculated in the left, right, RSL (relatively severe leg), and RML (relatively moderate leg), respectively. All the results were calculated after adjustment for age, *p < 0.05; **p < 0.01.

## Discussion

It is well known that the existence of a biomechanical alteration is examined in KOA. Lower-limbs biomechanical changes, including foot posture and anatomic axis angle of knee joint in KOA patients, could impair the biomechanical balance, cause inter-limb asymmetries, and consequently aggravate the KOA. It has been previously reported that the percentage of asymmetrical foot posture in KOA patients is greater than that in healthy subjects (Chen et al., 2020). In this study, it was proved that a more asymmetrical foot posture was closely associated with more severe KOA (higher K/L grade). Moreover, according to the asymmetry of foot posture categorized into normal, asymmetry, and severe asymmetry, we found that the ratio of normal in bilateral KOA patients was higher than that in unilateral ones. A previous study has shown that unilateral pain appeared to be associated with asymmetries in knee biomechanics (Creaby et al., 2012). Because of the interaction between knee biomechanics and foot posture, the patients with asymmetrical foot posture would tilt the body center of gravity to one side and probably present with unilateral pain. Therefore, the patients with unilateral pain had a higher ratio of asymmetry and severe asymmetry in foot posture than those with bilateral pain.

Human resting muscle (myofascial) tone that derives from its intrinsic molecular viscoelastic properties serves as an important factor for stability, and it should be considered among biomechanics (Masi and Hannon, 2008). GM and AT are attached to the calcaneus and play an important role in maintaining ankle stability, and they exhibit different properties at different severe degrees of KOA (Rutherford et al., 2011; Elbaz et al., 2017). Meanwhile, foot posture impacts leg muscle mass (Golightly et al., 2016), which could result in alterations of the muscle properties. Usually, the center of each muscle belly is recommended as the measurement site for muscle properties. In the present study, the measurement sites of LG and MG where cross-sectional areas of the GM are almost maximum were selected in accordance with the previous evidence (Feng et al., 2018; Huang et al., 2018). Meanwhile, it could be more capable of presenting a true situation for its characteristic properties (Schoenrock et al., 2018) when GM and AT stay in a relaxed state at 45° plantar flexion (Huang et al., 2018), and the most obvious difference in properties (tension stiffness and elasticity) of Achilles was observed at the 6 cm from calcaneus insertion (Morgan et al., 2018). As previously reported, slightly pronated foot postures were observed in healthy individuals (Redmond et al., 2008), and people with medial compartment KOA had increased pronated feet position (Levinger et al., 2010; Resende et al., 2016), which was significantly associated with increased calcaneus valgus angle (Ohi et al., 2017) and adduction moment of the knee (Signorile et al., 1995). It was suggested that the leg muscles showed different electromyographical activity in the subjects with different foot postures (Beaudreuil et al., 2009), and they also presented different leg muscles thicknesses (Ashnagar et al., 2019b). Obviously, foot posture had a great influence on leg muscles.

Results of this study have revealed that tone and stiffness in LG were greater than those in MG. This finding was compatible with the previous result observed in healthy subjects (Huang et al., 2018), and we thought that the difference between LG and MG should be more detectable in medial KOA patients because medial KOA representing varus alignment could cause lower limb and trunk mechanical changes that may overload the knee (Levinger et al., 2010), increase adduction moment of knee, and consequently increase the muscle tone of LG and decrease the tone of MG. It is well known that progression of medial knee osteoarthritis (KOA) has been associated with an increased knee adduction moment, and patients with advanced medial tibiofemoral OA have higher adduction moments (Sharma et al., 1998; Richards et al., 2018). Hence, there could be a negative correlation between the knee adduction moment and medial tibiofemoral joint width. When the medial tibiofemoral joint width becomes narrow, the increased varus angle of the knee will cause a stronger pull force toward LG, consequently leading to an increased tension (tone, stiffness) of LG. On the contrary, MG will show the decreased muscle tone and stiffness. Hence, LG has a greater muscle tone and stiffness than MG in patients with medial KOA, and there might be a significant relationship between adduction moment and muscle properties (tone and stiffness) of LG and MG. However, the relationship needs to be further identified in future studies. Furthermore, we found that an increased FPI (more pronated foot) was closely related to increases in the tone of ipsilateral LG. It should be because the valgus alignment decreased in a supination-to-pronation pattern in the foot, and increased varus angle would cause a stronger pull force toward LG, which consequently led to an increased tone of LG. Meanwhile, a significant linear relationship (an obviously positive correlation) was examined between FPI and tone of MG in the right side, and the same trend of near correlation to statistical difference was detected in the left side. This might be attributed to the fact that as LG and MG converged on AT and inserted into the calcaneus, increasing pronation (calcaneus varus angle increased) would exert a pull force to the AT, which would pull the MG and LG and increase the tone/tension in both of them. As their tone and tension were both increased, it was difficult to say if their difference in tone/tension was larger or smaller. Thus, no significant linear relationship was examined between FPI and D_-MLG(tone)_ in both left and right sides. Additionally, both LG and AT presented a greater tone in RSL than those in RLL. It seems to indicate that the leg with more severe symptomatic pain might be accompanied by alterations in mechanical properties in muscle and tendon. However, for the inter-muscle difference between MG and LG, we found no significant difference between RML and RSL. This might be because, in this study, both legs were categorized as RML and RSL according to the pain degree. More severe pain, in addition to biomechanical factors, caused more muscle tension, which resulted in a higher tone. However, in the ipsilateral leg, knee pain could affect both MG and LG; hence, D_-MLG(tone)_ between two legs did not vary much by pain. However, of note, although we could find that there were some correlations between asymmetrical FPI and asymmetry in GM and AT properties, the values of *R* ^2^ are in general low or very low (max is 0.176) according to the linear regression analysis. Considering that there must be a lot of factors influencing properties of muscle and tendon and foot posture, and the sample size may be a factor too, we thought relatively low values of *R* ^2^ might be acceptable, though the values of *R* ^2^ were in general low.

In the recent past, some studies have highlighted that some asymmetrical factors in limbs also played a very important role in the lower extremity function (Riskowski et al., 2012) and muscle strength (Lee et al., 2019). Most importantly, our finding demonstrated a significantly positive association between asymmetrical FPI scores and Asy_-AT(tension)_ and Asy_-AT(stiffness)_. In the light of the above-mentioned information, foot posture asymmetry could affect the Asy_-AT(tension)_ in KOA patients, especially people suffering from unilateral KOA.

GM, as well as hamstrings, was a two-joint muscle. For KOA patients, increased GM activity could provide active stiffness during early stance, potentially improving joint stability (Rutherford et al., 2011). Furthermore, as one of the risk factors for knee osteoarthritis, anterior cruciate ligament injury could cause selective atrophy of hamstring components, resulting in an imbalance of the medial and lateral hamstring activity (Lee et al., 2017). In contrast, the imbalance was associated with tibial rotation and the torsion angle of GM, which could directly influence knee stability (Jónasson et al., 2016). Notably, MG presented decreased activity in late stance, which was viewed as a strategy to reduce medial joint loading and being beneficial to KOA (Hubley-Kozey et al., 2006). Considering the effect of GM on the knee joint, it might play an important role in the progression of KOA. Additionally, it has been previously proved that patients with forefoot pathology presented a greater GM tightness than others (Malhotra et al., 2018), and GM played a role in the development of clinical pathology of the foot (Myerson, 2014).

Muscular tension could significantly reduce stability in standing posture and increase the risk of chronic joint overload or fall (Tassani et al., 2019), whereas muscle stiffness measures were proposed to evaluate individual muscle force and used to quantify local alternations of muscle impairments (Albin et al., 2020). In the present study, we found that the stiffness and tension of AT and the tone of LG in RSL were higher than those in RML in KOA patients; hence, softening the tone/tension LG and AT and decreasing the stiffness of AT could alleviate symptomatic pain and even reduce knee adduction moment for KOA. Moreover, findings from this study showed that inter-limbs foot posture and tension of AT were more asymmetrical in unilateral KOA patients than in patients with bilateral KOA, while it was suggested in a previous study (Chang et al., 2020) that the symmetric stiffness of the AT and GM was observed in both amateur basketball players and healthy subjects. Thus, asymmetrical muscle properties of GM and AT should be taken into consideration during the assessment and management of KOA. Furthermore, given that inter-limbs foot posture and tension of AT were more asymmetrical in unilateral KOA patients, and there was a positive association between asymmetrical FPI score and K/L grade, improving their asymmetries seemed to be critical. Insole could immediately correct foot posture, which should be a promising tool to modulate knee adduction moment for medial KOA (Sawada et al., 2017). However, because less asymmetrical foot posture and properties were detected between two limbs, improving properties of AT and LG, such as decreasing their tone and stiffness, should be high on the list of priorities when treating bilateral KOA patients. In addition, we found that increased FPI score (i.e., increasing pronation) might be closely related to the increased tone of the medial and lateral gastrocnemius, and there was a positive association between asymmetrical FPI score and K/L grade, which suggested that foot posture and its asymmetry served as important biomechanical factors associated with properties of AT and GM and severity of KOA. Meanwhile, considering the close relationship between foot posture, asymmetrical FPI, and muscle tone, we should not only correct the abnormal and asymmetrical foot posture but also take the properties of GM and AT into consideration during the treatment and rehabilitation of KOA patients. Most importantly, based on the findings in this study, we could deduce that, on the one hand, asymmetrical foot posture might be able to influence knee biomechanics by altering the muscle properties of AT and GM and due to changes of the axis of the lower extremity. On the other hand, improving their properties and inter-limbs muscle asymmetry might be a potential strategy to correct the asymmetrical foot posture and could even benefit KOA.

There were some limitations in the present study. Firstly, due to the lack of a healthy control group, it was unknown whether the muscle and tendon properties would be changed during KOA degenerative process, and we were not sure whether there was any difference in the symmetry of AT and GM properties between KOA and healthy subjects and whether the correlations existed in the health subjects or not. Secondly, foot posture was assessed only by FPI, which was a subjective scale; however, it would increase the degree of evidence for combining an objective method. Thirdly, taking into account the fact that the most common KOA was the medial type in China (Sun et al., 2019), we only included the subjects affected by predominantly medial compartment OA, which might lead to a bias; hence, we should explore the findings in future studies.

## Conclusion

The stiffness and tension of AT and the tone of LG in RSL were higher than those in RML in KOA patients, and inter-limbs foot posture and tension of AT were more asymmetrical in unilateral KOA patients than those in patients with bilateral KOA. Moreover, a significant correlation was found between the FPI score and the tone of GM, and the asymmetry of FPI was closely associated with the asymmetry of AT. Additionally, there was a positive relationship between asymmetrical FPI score and K/L grade. Therefore, foot posture, properties of GM and AT, and their symmetries were clinically relevant in KOA patients, which might provide a novel insight into the treatment and rehabilitation of KOA.

## Data Availability

The data used to support the findings of this study are available from the corresponding author upon request.
