# Supplementary material for: Comparison of the Asymmetries in Foot Posture and Properties of Gastrocnemius Muscle and Achilles Tendon Between Patients With Unilateral and Bilateral Knee Osteoarthritis
Source: Front Bioeng Biotechnol. 2021 Oct 14;9:636571. doi: 10.3389/fbioe.2021.636571 (PMC8561212; doi:10.3389/fbioe.2021.636571)
Supplement: Supplementary file 1 [file DataSheet1.docx]

**Supplemental Appendix S1**

**Method 1**

$Asy(\%)=\left( \frac{V_{larger}-V_{lower}}{V_{larger}} \right)\times100$

Achilles tendon asymmetry index (*Asy_-AT_*) was calculated using this equation.

*Asy:* asymmetery index; *V_larger_*: larger value; *V_lower_*: lower value.


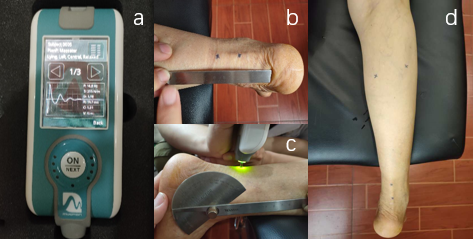


**eFigure 1** Example of measurement performed with the portable digital palpation device (MyotonPRO).

a: MyotonPRO device; b:measurement points of Achilles tendon; c: assessing for Achilles tendon; d: measure points of medial and lateral of gastrocnemius muscle.

a b c

**eFigure 2** comparison of muscle properties in lateral and medial of gastrocnemius.

MG: medial of gastrocnemius; LG: lateral of gastrocnemius; compared to LG, *: P < 0.05; **: P < 0.01.

**eTable 1** Comparison of differences in muscle properties between medial and lateral gastrocnemius.

| Groups | D_-MLG_ | | |
| --- | --- | --- | --- |
|  | D_-MLG(tone)_ | D_-MLG(stiffness)_ | D_-MLG(elasticity)_ |
| RHL | 1.51 $\pm$ 0.96 | 26.65 $\pm$ 21.25 | 0.30 $\pm$ 0.26 |
| RLL | 1.27 $\pm$ 0.84 | 28.66 $\pm$ 22.47 | 0.32 $\pm$ 0.29 |
| P | 0.114 | 0.776 | 0.968 |

D_-MLG:_ differences in muscle properties between medial and lateral gastrocnemius in the ipsilateral limb; D_-MLG(tone)_: difference in tone; D_-MLG(stiffness)_: difference in stiffness; D_-MLG(elasticity)_: difference in elasticity; RSL: relatively severe leg; RML relatively moderate leg.
